# CA19-9-producing lung metastasis after surgery for papillary thyroid carcinoma: report of a case

**DOI:** 10.1007/s00595-013-0820-1

**Published:** 2014-01-10

**Authors:** Emi Yamaguchi, Yoshinari Makino, Takashi Sato, Masaaki Uchida, Yuji Harada, Riruke Maruyama

**Affiliations:** 1 Department of Surgery, Izumo-City General Medical Center, 613 Nadabun-cho, Izumo, Shimane 691-0003 Japan; 2 Department of Surgery, Matsue Seikyo Hospital, Matsue, Shimane 690-8522 Japan; 3 Department of Pathology, Organ Pathology Unit, Shimane University Faculty of Medicine, Izumo, Shimane 693-8501 Japan; 4 Laboratory of Surgical Pathology, Shimane University Hospital, Izumo, Shimane 693-8501 Japan

**Keywords:** Papillary thyroid carcinoma, CA19-9-producing tumor, Metachronous metastasis

## Abstract

Measuring tumor marker levels following cancer treatment can be useful. Although serum thyroglobulin is a useful marker after total thyroidectomy for papillary thyroid carcinoma (PTC), it is not a reliable marker for patients with a high titer of anti-thyroglobulin antibodies or when transformation to undifferentiated carcinoma has occurred. The female patient in this case report underwent total thyroidectomy and oral I-131 therapy for PTC at the age of 47 years, followed by cervical lymph node and lung resections for metastases, 3 and 11 years later, respectively. She also received oral I-131 therapy and external beam radiotherapy for mediastinal lymph node metastases. The lymphadenopathy lesions progressed and multiple lung metastases were detected when she was 61 years of age. She died at the age of 62 years. The serum CA19-9 level had gradually increased in association with enlargement of the recurrent lesions and immunostaining of CA19-9 in the pulmonary metastasis was intense. Thus, we consider that measuring the level of serum CA19-9 is an effective tool for evaluating disease status after surgery for PTC.

## Introduction

Tumor markers are important tools for screening, monitoring therapeutic effects, and detecting recurrence early, after radical treatment for malignant disease. Although serum thyroglobulin is a useful marker in patients who have undergone total thyroidectomy for papillary thyroid carcinoma (PTC), it is not a reliable marker in those with a high titer of anti-thyroglobulin antibodies or when transformation to undifferentiated carcinoma has occurred. We report a case of carbohydrate antigen 19-9 (CA19-9)-producing metastasis of PTC in a patient with an elevated serum CA19-9 level.

## Case report

### Initial surgery and metachronous lymph node metastasis

**Fig. 1:**
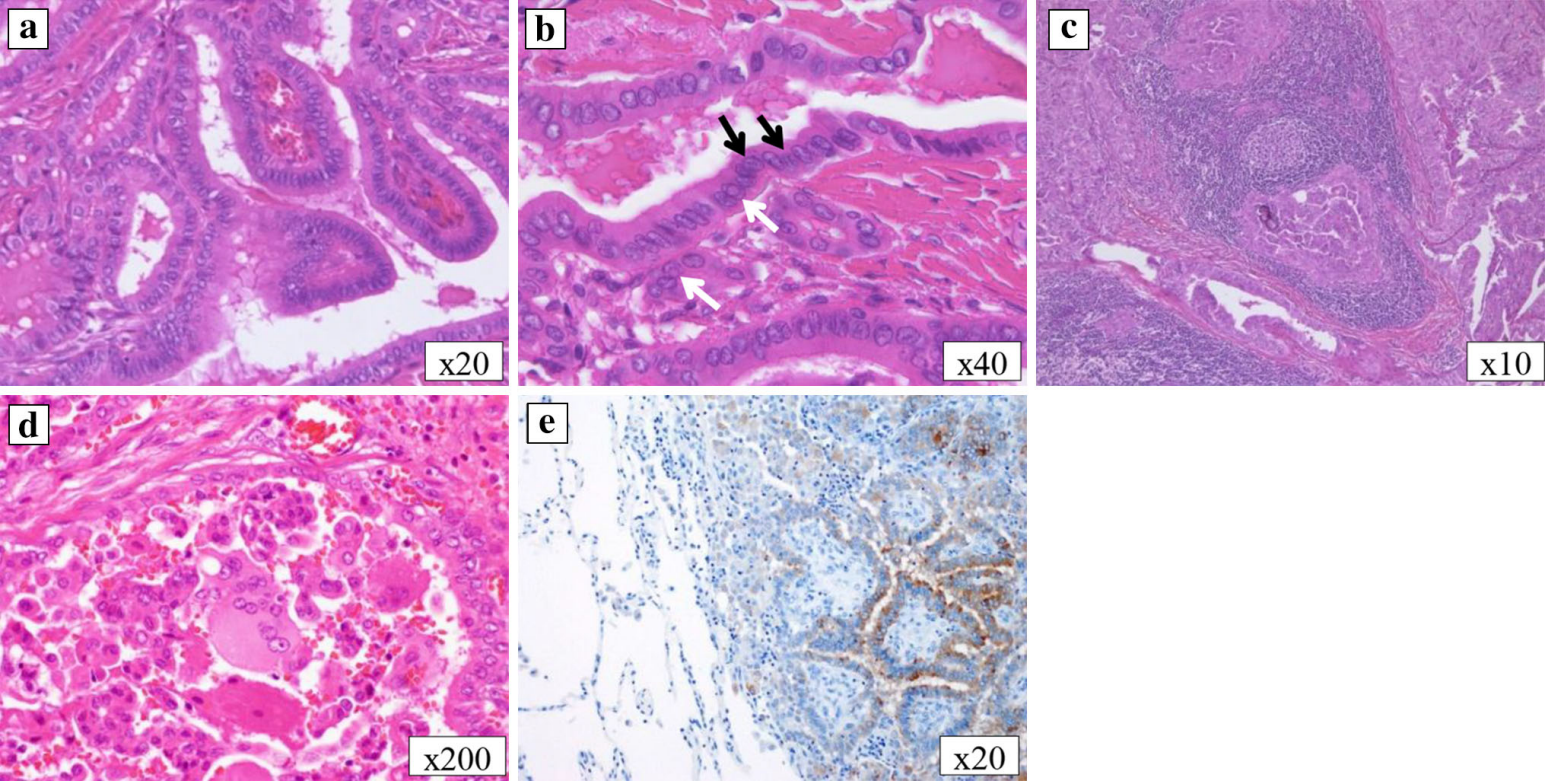
Microscopic findings of the thyroid, lymph node, and lung tumors. In the primary thyroid lesion, the tumor cells exhibited a typical papillary structure (**a**), nuclear grooves (*black arrow*) and intranuclear cytoplasmic inclusion (*white arrow*) (**b**). The lymph node was diagnosed as metastasis of papillary thyroid carcinoma (**c**). There were multinucleated cells; however, no cellular atypia was observed within the lung tumor (**d**). Thyroglobulin was stained within the lung tumor (**e**). **a**–**d** H&E staining, **e** thyroglobulin staining

The patient was a 62-year-old woman with bronchial asthma treated with pharmacotherapy. At the age of 47 years, she had undergone total thyroidectomy and en-bloc resection of the tracheal cartilage for thyroid carcinoma with direct tracheal invasion and bilateral cervical and superior mediastinal lymph node dissection. Histological analysis revealed tumor cells with a typical papillary structure, with nuclear grooves and intranuclear cytoplasmic inclusion, leading to a diagnosis of PTC (Fig. 1a, b). Some areas of the specimen exhibited a micropapillary pattern with an ill-defined stromal axis or solid nest pattern. These findings suggested malignancy of a higher grade than typical PTC. Tumor invasion into the surrounding muscle and metastases in the cervical and mediastinal lymph nodes were also identified. The TNM classification was T4aN1bM0, stage IVA. About 6 months after surgery, oral I-131 therapy was administered at a dose of 150 mCi when radioiodine (RI) accumulation was detected in the mediastinum. She was then treated with thyroid-stimulating hormone (TSH) suppression therapy. Excisional biopsy of one cervical lymph node was performed 2 years and 6 months after surgery and the histological diagnosis was metastasis of PTC (Fig. 1c). The patient was then referred to our hospital for outpatient follow-up.

### Lung and superior mediastinal lymph node metastases

Positron emission tomography (PET), done 11 years and 4 months after the initial surgery, revealed ^18^F-fluorodeoxyglucose (FDG) accumulation in a left pulmonary mass and the superior mediastinum. Thus, we performed thoracoscopic partial left lower lobectomy. Histologically, the diagnosis was metastasis of PTC. There were multinucleated cells in the surgical specimen, without cellular atypia, suggestive of anaplastic carcinoma (Fig. 1d). However, there was no structural atypia; therefore, anaplastic carcinoma was excluded. We diagnosed FDG accumulation in the superior mediastinal area, reflecting lymph node metastasis of PTC. Oral I-131 therapy (150 mCi) was administered at the patient’s request. Scintigraphy revealed minimal RI accumulation after RI therapy, and there were no remarkable changes. About 1 year after pulmonary resection, PET-CT revealed that the mediastinal lymphadenopathy had become more pronounced; however, there were no metastatic masses in the lung fields. The mediastinal lymph nodes were treated with external beam radiotherapy (60 Gy/30 fr) at the patient’s request. As there were no remarkable changes within the mediastinum, TSH suppression therapy alone was administered. By 14 years and 4 months after the initial surgery, multiple lung metastases and supraclavicular lymphadenopathy were noted; however, the mediastinal lymphadenopathy had not worsened. The patient had no subjective symptoms and the follow-up was uneventful. However, the metastatic lesions subsequently enlarged, and the patient died 15 years and 2 months after the initial surgery.

### Blood tests and immunohistological examinations (Figs. 2, 3)

The serum thyroglobulin level increased twice: initially, during discontinuation of thyroid hormone administration before the oral I-131 therapy, and then during external beam radiotherapy. However, it was not increased when the first lung metastasis was found or during progression of the mediastinal or multiple lung metastases. At the time, the initial pulmonary mass was visualized on imaging, and all tumor markers for primary lung carcinoma, namely, carcinoembryonic antigen (CEA), sialyl LewisX, squamous cell carcinoma-related antigen, cytokeratin 19 fragment, neuron-specific enolase, and pro-gastrin-releasing peptide, were negative. Although no malignant tumors, except for the pulmonary mass, were detected on imaging studies, including upper and lower gastrointestinal endoscopic imaging, only the serum CA19-9 level was elevated, at 41.6 U/ml. By 6 months after pulmonary resection, the CA19-9 level had decreased to 28.4 U/ml. Following pulmonary resection, serial CA19-9 levels were above the normal limits, corresponding to enlargement of the superior mediastinal lymph node and multiple lung metastases. Intense staining was observed in the cytoplasm of the pulmonary lesions with immunostaining of CA19-9, although there was no staining in the primary thyroid lesion or cervical lymph node metastasis (Fig. 3a–c). Furthermore, there were multinucleated cells, without cellular atypia inside the lung lesion (Fig. 1d), while immunostaining of thyroglobulin was positive within the lung tumor (Fig. 1e).

**Fig. 2:**
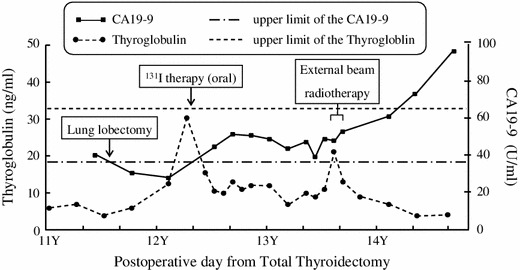
Changes in the serum CA19-9 and thyroglobulin levels. The CA19-9 level increased when the lung metastasis was diagnosed. The level normalized following resection of the metastases, then increased again with enlargement of the superior mediastinal lymph node and multiple lung metastases. The serum thyroglobulin level increased when TSH suppression therapy was discontinued before the administration of oral I-131 therapy and during external beam radiotherapy; however, no elevation of the thyroglobulin level was observed during the enlargement of the mediastinal and multiple lung metastases

**Fig. 3:**
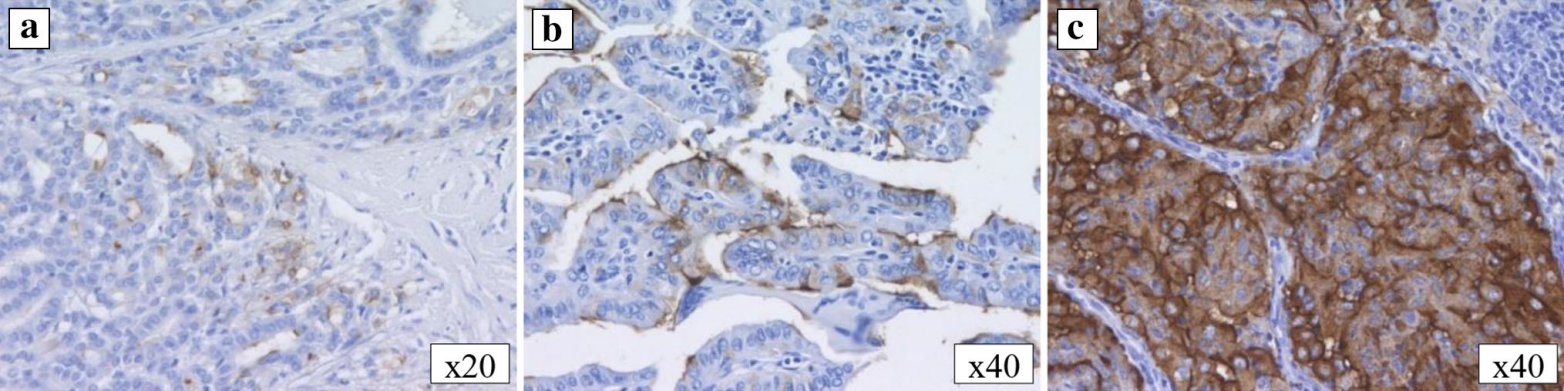
Microscopic findings (CA19-9 staining) of the thyroid, cervical lymph node, and lung tumors. The primary thyroid tumor (**a**) and cervical lymph node (**b**) were negative for CA19-9, whereas the lung tumor (**c**) was positive

## Discussion

Tumor markers are important for cancer screening, therapeutic evaluation, and early detection of recurrence. CEA and calcitonin are widely known as tumor markers for medullary thyroid carcinoma, whereas serum thyroglobulin has been established as an effective tumor marker for evaluating disease status after total thyroidectomy and as an indicator of recurrence, although its distribution is affected by the presence of anti-thyroglobulin antibodies. Thus, serum thyroglobulin is not a reliable marker for patients with a high titer of anti-thyroglobulin antibodies or in cases of transformation to undifferentiated carcinoma. In the present case, the serum thyroglobulin level increased twice. Initially, we thought that the increase during oral I-131 therapy was caused by elevation of the TSH during the discontinuation of thyroid hormone administration, as the serum thyroglobulin level decreased at the restart of TSH suppression therapy. At the second instance of thyroglobulin elevation, we speculated that the change in the serum thyroglobulin level observed during external beam radiotherapy was due to collapse of the tumor cells, although no remarkable reductions in the metastatic lesions were noted. However, the serum thyroglobulin level did not increase when lung metastasis was found or during enlargement of the mediastinal and multiple lung metastases. Therefore, in this case, the serum thyroglobulin level was not a useful tumor marker.

CA19-9 levels are known to be elevated in patients with cancers of the digestive system; however, they rarely increase in patients with thyroid carcinoma. A search of PubMed and ICHUSHI (Japan Medical Abstracts Society) in 2012 using the keywords, “papillary thyroid carcinoma” and “CA19-9,” revealed only four Japanese papers with detailed cases reports [1–4]. Hashimoto et al. [1] examined 111 patients with thyroid carcinoma and reported that 58 % exhibited positive immunohistochemical staining for CA19-9 at the time of diagnosis, although only 5.3 % had an elevated level of serum CA19-9. Vierbuchen et al. [5] reported positive immunostaining for CA19-9 in PTC tissues, suggesting CA19-9 production in PTC. In our patient, the CA19-9 level was elevated before excision of the lung metastasis and decreased to within the normal limits thereafter. Furthermore, the histopathological diagnosis of the lung mass was PTC and immunostaining was positive for CA19-9. We speculated that CA19-9 was produced by the PTC cells in the metastatic lung lesion. The results of thyroglobulin staining of the first lung metastases supported our speculation. On the other hand, the primary thyroid carcinoma and lymph node metastases were not stained with CA19-9 on immunohistochemistry. Matsuura et al. [3] reported that the serum level of CA19-9 is a useful marker for monitoring the growth and/or recurrence of PTC when a high serum level of CA19-9 is observed. Nomizu et al. [6] also reported low sensitivity of CA19-9 in patients with thyroid carcinoma. Therefore, in the present case, we thought that low sensitivity of CA19-9 was why immunostaining of CA19-9 was negative in the primary thyroid lesion and lymph node metastases and serum CA19-9 was not detected, even though PTC lesions can produce CA19-9. Thus, in the present case, the serum CA19-9 level was useful for monitoring recurrence and progression of the metastatic lesions, being a more effective tumor marker than the serum thyroglobulin level.

Serum CA19-9 levels have been reported to increase in patients with undifferentiated thyroid carcinoma [7]. Ogawa et al. [4] reported that serum CA19-9 levels increased significantly during the transformation of differentiated thyroid carcinoma into undifferentiated carcinoma. In the present case, the resected metastatic lung lesion was diagnosed as PTC and immunostaining for CA19-9 and thyroglobulin in this lesion was positive. Therefore, we speculated that the PTC produced the CA19-9. Although we were unable to perform an autopsy, the PTC lesion may have transformed into undifferentiated carcinoma during exacerbation of the multiple lung metastases and progression of the mediastinal metastases. The thyroid carcinoma cells may have strengthened to produce CA19-9, so we reviewed the possibility that the undifferentiated carcinoma produced thyroglobulin and the PTC transformed into undifferentiated thyroid carcinoma.

There are two theories regarding the transformation of PTC into undifferentiated carcinoma, including the effects of oral I-131 therapy and external beam radiotherapy and the natural history of PTC. Crile et al. [8] reported that the anaplastic transformation of PTC is affected by the administration of oral or external radiation therapy. On the other hand, Harada et al. reported that anaplastic transformation is part of the natural course of PTC, based on the results of 27 autopsies among 518 cases of thyroid cancer. Osumi et al. [9] also reported a correlation between differentiated thyroid carcinoma and the administration of oral or external radiation therapy in five patients. Transformation into undifferentiated carcinoma was found in three of these patients, who did not receive radiation therapy. Another two patients did not exhibit anaplastic transformation and did receive radiation therapy. Therefore, the PTC in our patient may have undergone anaplastic transformation as a result of a long natural course after the primary surgery or from the administration of oral or external radiation therapy.

We considered the possibility of undifferentiated thyroid carcinoma and the production of thyroglobulin. Venkatesh et al. [10] performed immunostaining of thyroglobulin in 30 of 112 cases of undifferentiated thyroid carcinoma. They reported no staining with thyroglobulin in 90 % of the cases and that, although the remaining 10 % had staining with thyroglobulin, the positive cells accounted for only 1–25 % of the tumor cells. Wiseman et al. [11] also reported that anaplastic transformed thyroid carcinoma cells do not have an ability to produce thyroglobulin based on the results of a microarray analysis of 12 cases of anaplastic transformed thyroid carcinoma.

We speculated that our patient had a high titer of anti-thyroglobulin antibodies and that the tumor lost its ability to produce thyroglobulin because it transformed into undifferentiated carcinoma when the multiple lung and mediastinal metastases developed. However, we considered that the PTC cells had the ability to produce thyroglobulin, at least at the time of the first lung metastasis, based on the results of histological diagnosis of lung metastasis and immunostaining of thyroglobulin. Thus, the serum thyroglobulin level is not an effective marker for the detection of recurrence and/or the therapeutic evaluation of long-term survival in PTC patients who have undergone surgery, in patients with a high titer of anti-thyroglobulin antibodies, or those whose tumor cells have transformed into undifferentiated carcinoma. The serum CA19-9 level was a much more useful tumor marker in the present case. Currently, PET examinations are done to assess malignant disease; however, as only a limited number of facilities in Japan can perform expensive PET scans, it is not yet a suitable screening test. Conversely, blood tests for tumor markers are minimally invasive and can be useful.

In conclusion, the serum CA19-9 concentration may become an effective tool for evaluating disease status after surgery for PTC. Measuring the level of serum CA19-9 is meaningful in patients with positive anti-thyroglobulin antibodies and in those with transformation of PTC to undifferentiated carcinoma. Early detection and treatment can be provided and better prognoses can be achieved with this diagnostic tool.
